# Risks of Treated Insomnia, Anxiety, and Depression in Health Care-Seeking Physicians: A Nationwide Population-Based Study

**DOI:** 10.1097/MD.0000000000001323

**Published:** 2015-09-04

**Authors:** Charles Lung-Cheng Huang, Shih-Feng Weng, Jhi-Joung Wang, Ya-Wen Hsu, Ming-Ping Wu

**Affiliations:** From the Department of Psychiatry, Chi Mei Hospital; Department of Social Worker, Chia Nan University of Pharmacy and Science (CL-CH); Department of Medical Research, Chi Mei Hospital; Department of Hospital and Health Care Administration, Chia Nan University of Pharmacy and Science (S-FW, J-JW, Y-WH); Division of Urogynecology and Pelvic Floor Reconstruction, Department of Obstetrics and Gynecology, Chi Mei Hospital; and Center of General Education, Chia Nan University of Pharmacy and Science, Tainan, Taiwan (M-PW).

## Abstract

High occupational stress and burnout among physicians can lead to sleep problems, anxiety, depression, and even suicide. Even so, the actual risk for these behavioral health problems in health care-seeking physicians has been seldom explored. The aim of this study was to determine whether physicians have higher odds of treated insomnia, anxiety, and depression than the normal population.

This is a nationwide population-based case–control study using the National Health Insurance Research Database in Taiwan for the years 2007 to 2011. Physicians were obtained from the Registry for Medical Personnel in 2009. Hospital physicians who had at least 3 coded ambulatory care claims or 1 inpatient claim with a principal diagnosis of insomnia, anxiety, or depression were identified. A total of 15,150 physicians and 45,450 matched controls were enrolled. Odd ratios (ORs) of insomnia, anxiety, and depression between physicians and their control counterparts were measured.

The adjusted ORs for treated insomnia, anxiety, and depression among all studied physicians were 2.028 (95% confidence interval [CI], 1.892–2.175), 1.103 (95% CI, 1.020–1.193), and 0.716 (95% CI, 0.630–0.813), respectively. All specialties of physicians had significantly higher ORs for treated insomnia; among the highest was the emergency specialty. The adjusted ORs for treated anxiety among male and female physicians were 1.136 (95% CI, 1.039–1.242) and 0.827 (95% CI, 0.686–0.997), respectively. Among specialties, psychiatry and “others” had significantly higher risks of anxiety. Obstetrics and gynecology and surgery specialties had significantly lower risks of anxiety. The adjusted ORs for treated depression among physicians in age groups 35 to 50 years and >50 years were 0.560 (95% CI, 0.459–0.683) and 0.770 (95% CI, 0.619–0.959), respectively. Those in the psychiatry specialty had significantly higher risks of depression; internal and surgery specialties had significant lower risks of depression.

Hospital physicians have lower odds of treated depression than the general population, although they have higher odd of treated insomnia and anxiety. Undertreatment was noted in some sex, age, and specialty subgroups of physicians. Additional studies are needed to determine how to eliminate barriers to their use of psychiatry resources.

## INTRODUCTION

Practicing medicine is stressful to many physicians due to excessive workloads, extended working hours, high levels of time pressure, and restricted autonomy. Burnout, viewed as the exhaustion of physical or emotional strength as a result of prolonged stress or frustration, has been detected in physicians.^1–4^ The high level of occupational stress and burnout among physicians can lead to sleep problems,^5–7^ anxiety,^6,8–11^ depression,^8,9,11–16^ and even suicide.^4,14,17–21^

As for Taiwan, in the face of increased pressure resulting from heavy clinical workloads and litigation, academic competition, regulations and limitations regarding National Health Insurance payments, and tense doctor–patient relationships, etc, physicians’ working conditions have become highly stressful and exhausting in recent years. Therefore, more and more physicians in Taiwan feel frustrated and burned out in their jobs, and several incidents of depression and suicide among physicians have attracted public attention. For example, a recent Taiwanese study reported that 13.3% of physicians had reached the level of clinical depression, which was higher than that found in the general population (3.7%) of Taiwan.^15^

Stress, burnout, and subsequent behavioral health problems among physicians are becoming a worldwide public health issue.^22^ Physician wellness might not only avail the physicians themselves, it could also be crucial to the providing of high-quality health care^23^; however, studies suggest that doctors are not enthusiastic at caring to their wellness needs or seeking help from other professionals.^24,25^ It was noted that physicians seek help to a lesser degree and later in the course of disease than do other groups,^26–28^ and they appear to be more reluctant to seek help for behavioral health or psychologic problems.^4,20,29–31^

An increasing body of research suggests physicians have higher risks of anxiety/depression/sleep disorder that require clinical treatment.^5,10,13,14^ There appears to be inadequate attention given to these psychiatric health problems and hesitation to seek help among physicians due to concerns about confidentiality or other reasons^4,20,22,23,32^; however, there are few studies on the actual risk for these common psychiatric problems in health care-seeking physicians other than questionnaire surveys.^30^ Most of these studies have been based on self-reported surveys using rating scales for anxiety/depression/sleep disorder rather than physician-confirmed diagnoses. In addition, the sample sizes in most studies were limited. In this study, using a nationwide population-based database containing data on enrollees in the National Health Insurance program in Taiwan, our objective was to verify the hypotheses that health care-seeking physicians may have higher odds of treated anxiety/depression/insomnia due to higher risks of these psychiatric problems than the general population; or that health care-seeking physicians may have similar or even lower odds of treated anxiety/depression/insomnia due to some reasons such as reluctance.

## MATERIALS AND METHODS

### Study Design

We constructed a nationwide case–control study in attempt to compare the risk of treated insomnia, anxiety, and depression between physicians and control counterparts. We consulted with the institutional review board of Chi Mei Medical Center and obtained a formal written waiver for the need of ethics approval (No. 10206-E06).

### Data Sources

In this study, the medical claims data were retrieved from the Taiwan's National Health Insurance Research Database (NHIRD). The data consist of outpatient (ambulatory) care and inpatient care records and the registration files of the Taiwan NHIRD, which is a universal health care system that covers 99% of the country's population of 23.3 million. The confidentiality of individuals was protected using encrypted personal identification to avoid the possibility of the ethical violations related to the data. International Classification of Diseases, Ninth Revision, Clinical Modification (ICD-9-CM) codes are applied for clinical diagnoses and procedures, details of prescribed drugs, dates of admission and discharge, and basic sociodemographic information, including sex and date of birth. The validity of the NHIRD has been confirmed because hundreds of studies have been published using the database.^33^ Information on medical personnel (including physicians and other health care providers) is also available and includes licensed date, specialty, work area, hospital level, types of employment, and encrypted identification number, which can be linked to the aforementioned claims data. All medical expenses for diabetes mellitus (DM), hypertension (HTN), coronary artery disease (CAD), hyperlipidemia, insomnia, anxiety and depression are covered by National Health Insurance.

### Selection of Cases and Controls

**FIGURE 1:**
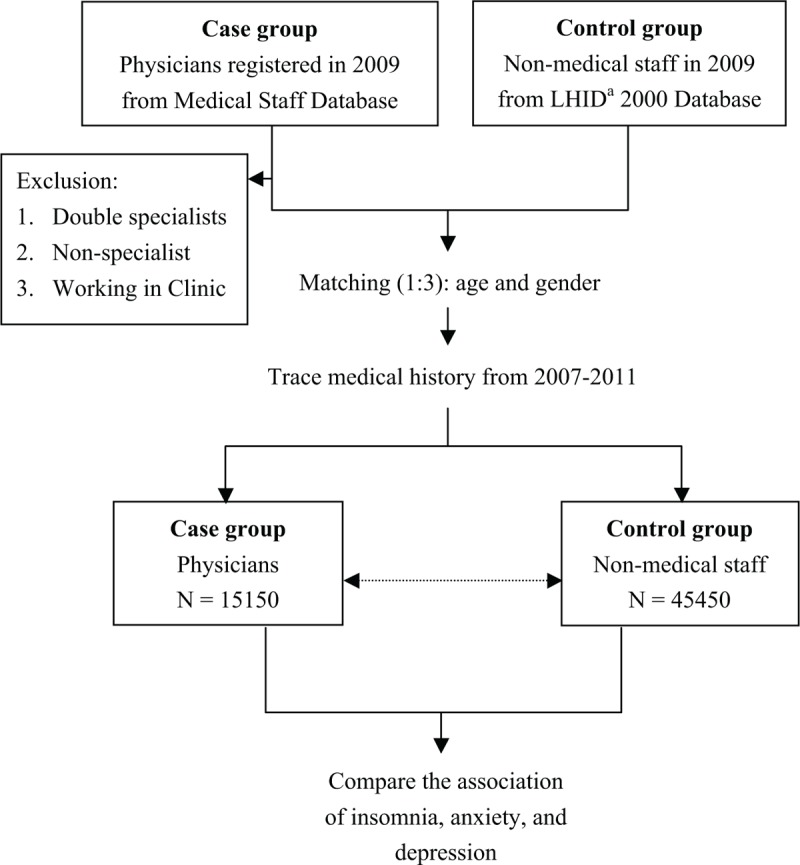
Flow diagram showing the patient population for evaluation in the study. ^a^LHID2000 = Longitudinal Health Insurance Database 2000.

In this study, the data on physicians were obtained from the Registry for Medical Personnel, which contains all registered medical staff in 2009. Then, we excluded physicians who were not specialists, double specialists (eg, a physician with both boards of internal medicine and emergency medicine certifications), and working in the clinic (Figure 1). The reason we excluded double specialists was the difficulty in assigning them to subgroup comparison. Specialists who work in the clinics were excluded because the working environment and demands of clinics and hospitals were quite different. For the control counterparts, we selected 3 matches (nonmedical staff) per case from the Longitudinal Health Insurance Database 2000, which contains all claims data of 1 million (4.34% of the total population) Registry for Beneficiaries who were randomly selected in 2000 (Figure 1) of the NHIRD. Any individual who was once a beneficiary of the National Health Insurance program in 2000 would be included in the registry, not only sick patients. There are no significant differences in age, sex, or health care costs between the Longitudinal Health Insurance Database 2000 and all National Health Insurance enrollees. Controls were matched with cases in terms of age, birth year, and sex (Figure 1).

We linked to the diagnostic codes through the inpatient and ambulatory care claims databases of the National Health Insurance. Common comorbidities that may affect anxiety and depression were defined as follows: DM (ICD-9CM code 250), HTN (ICD-9CM codes 401–405), CAD (ICD-9CM code 410–414), and hyperlipidemia (ICD-9-CM codes 272). These comorbidities were counted if they occurred in >3 ambulatory care claims coded or 1 inpatient claim from 2007 to 2011.

### Exposure Assessment

In this study, we attempted to compare the risk of treated insomnia, anxiety, and depression between physicians and control counterparts. Insomnia was defined as follows, using ICD-9CM codes: transient disorder of initiating or maintaining sleep (307.41); persistent disorder of initiating or maintaining sleep (307.42); and unspecified insomnia (780.52). Anxiety was defined as follows, with ICD-9-CM codes: anxiety, dissociative and somatoform disorders (300, excluding 300.4); with predominant disturbance of other emotions (309.2); adjustment disorder with disturbance of conduct (309.3); adjustment disorder with mixed disturbance of emotions and conduct (309.4). The ICD-9-CM code 300 includes common anxiety disorders such as generalized anxiety disorder, posttraumatic stress disorder, and obsessive-compulsive disorder. Depression was defined as follows, using ICD-9-CM codes: major depressive disorder, single episode (296.2); major depressive disorder, recurrent episode (296.3); bipolar I disorder, most recent episode (or current) of depression (296.5); depressive disorder, not elsewhere classified (311); dysthymic disorder (300.4); adjustment disorder with depressed mood (309.0); and prolonged depressive reaction (309.1). All the above exposure outcomes were found when tracing the 2007 to 2011 medical history of who had at least 3 outpatient service claims in 1 year, or at least 1 inpatient hospitalization claim (Figure 1).

### Physician Subgroups Analysis

We further analyzed the subgroups of physicians based on specialty, age, sex, area, comorbidities, and hospital level (Figure 1). Specialists who practice in emergency and critical care (ie, internal medicine, surgery, obstetrics and gynecology (OBS/GYN), pediatrics, emergency medicine, psychiatry, orthopedics, and anesthesiology) may have a higher risk of insomnia, anxiety, and depression due to greater work demands. Therefore, we divided physicians into 9 subgroups for comparison: internal medicine, surgery, OBS/GYN, pediatrics, emergency medicine, psychiatry, orthopedics, anesthesiology, and others. The “others” group included specialties that are not included in preselected ones (eg, neurology, rehabilitation, family medicine, dermatology, ENT, ophthalmology, radiology, urology, pathology, and nuclear medicine).

### Statistical Analyses

We used conditional logistic regression to obtain the odds ratios (ORs) of treated insomnia, anxiety, and depression between physicians and control counterparts (as the reference group), controlling for potential confounders such as DM, HTN, CAD, hyperlipidemia and area. All analyses were stratified by age, sex, and specialty, and were conducted with SAS 9.4 for Windows (SAS Institute Inc., Cary, NC). A two-tailed *P* < 0.05 was considered significant.

## RESULTS

A total of 15,150 physicians and 45,450 matched controls were enrolled. There were no significant differences in age, sex, and CAD between the 2 groups (Table 1). After controlling for DM, HTN, CAD, and hyperlipidemia, the adjusted OR for treated insomnia among all physicians was 2.028. All specialties of physicians had significantly higher ORs for treated insomnia; among the highest was the emergency specialty (OR = 5.088) (Table 2).

**Table 1:**
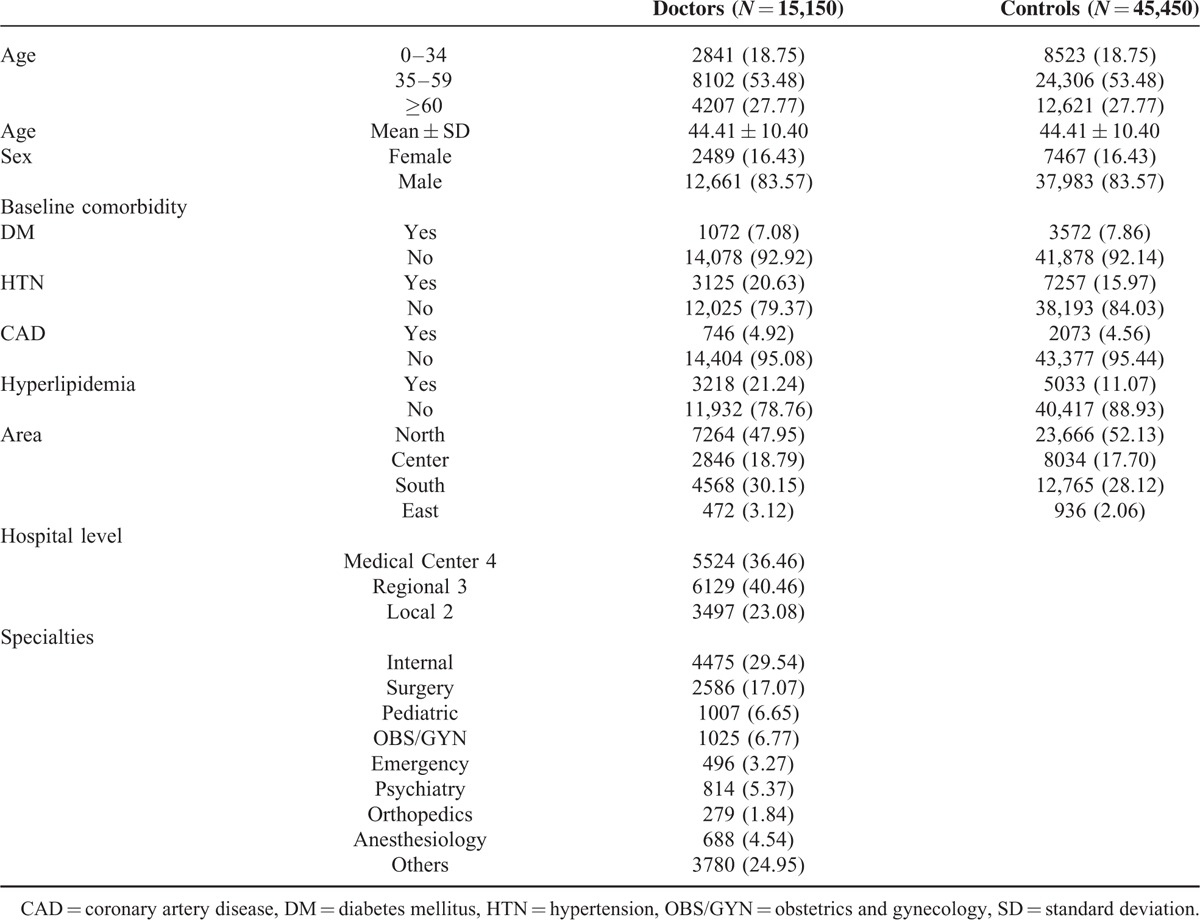
Demographic Characteristics and Comorbid Medical Disorders of Physicians and Comparison Group Patients

**TABLE 2:**
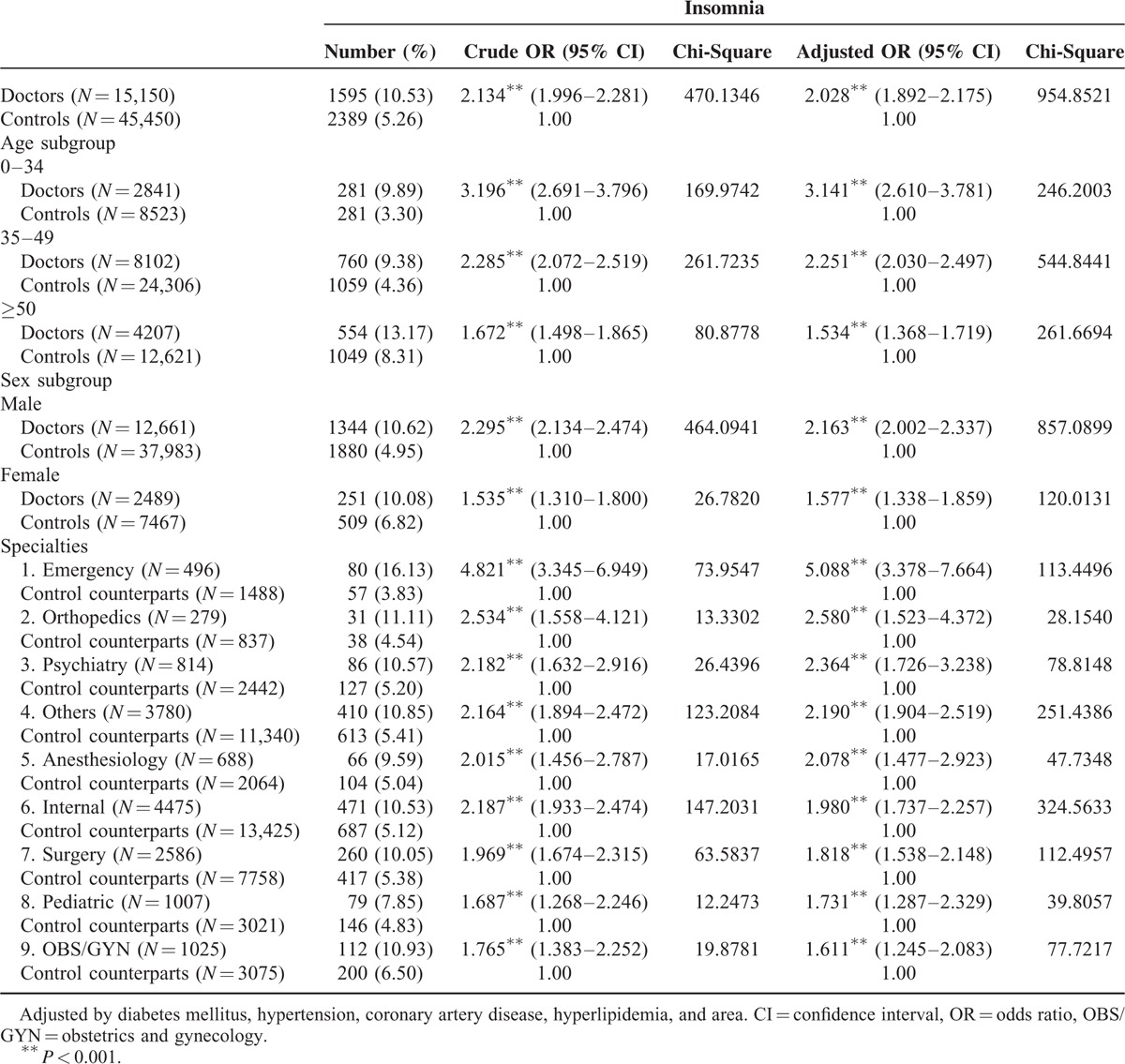
Stratified ORs for Insomnia by Age, Sex, and Specialty of Physicians

After controlling for DM, HTN, CAD, and hyperlipidemia, the adjusted OR for treated anxiety among all physicians was 1.103. Male physicians had a significantly higher OR (1.136) and female physicians had a lower OR (0.827) for treated anxiety. The psychiatry and “other” specialties had significantly higher ORs for treated anxiety (3.073 and 1.189, respectively). On the contrary, the OBS/GYN and surgery specialties had significantly lower ORs for treated anxiety (0.719 and 0.641, respectively) (Table 3).

**TABLE 3:**
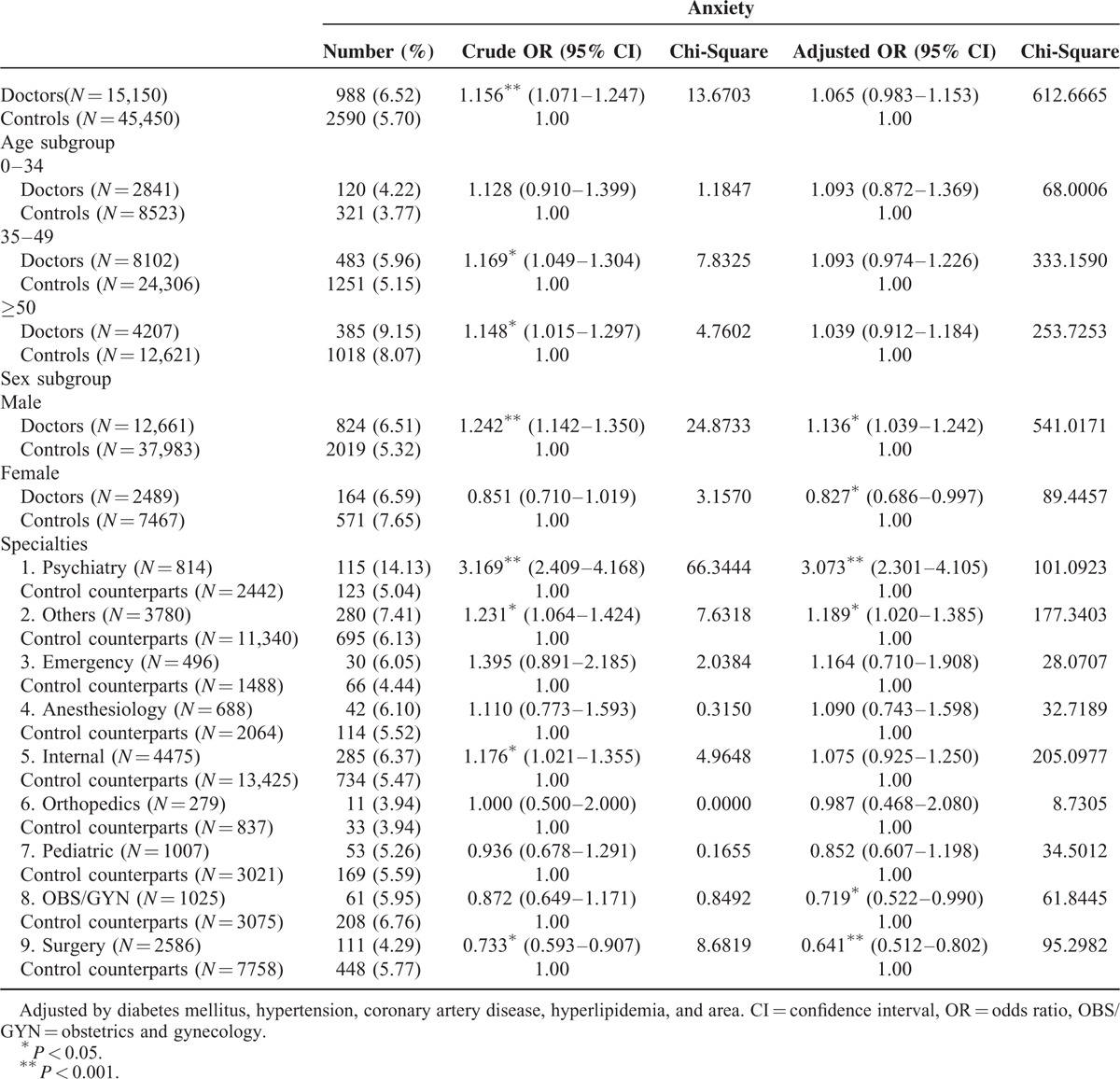
Stratified ORs for Anxiety by Age, Sex, and Specialty of Physicians

After controlling for DM, HTN, CAD, and hyperlipidemia, the adjusted OR for treated depression among all physicians was 0.716. There was no significant difference between males and females. Physicians in the age groups of 35 to 50 and >50 years had significantly lower ORs for treated depression (0.560 and 0.770, respectively). The psychiatry specialty had a significantly higher OR (3.060) for treated depression; the internal medicine and surgery specialties had significantly lower ORs for treated depression (0.551 and 0.328, respectively) (Table 4).

**TABLE 4:**
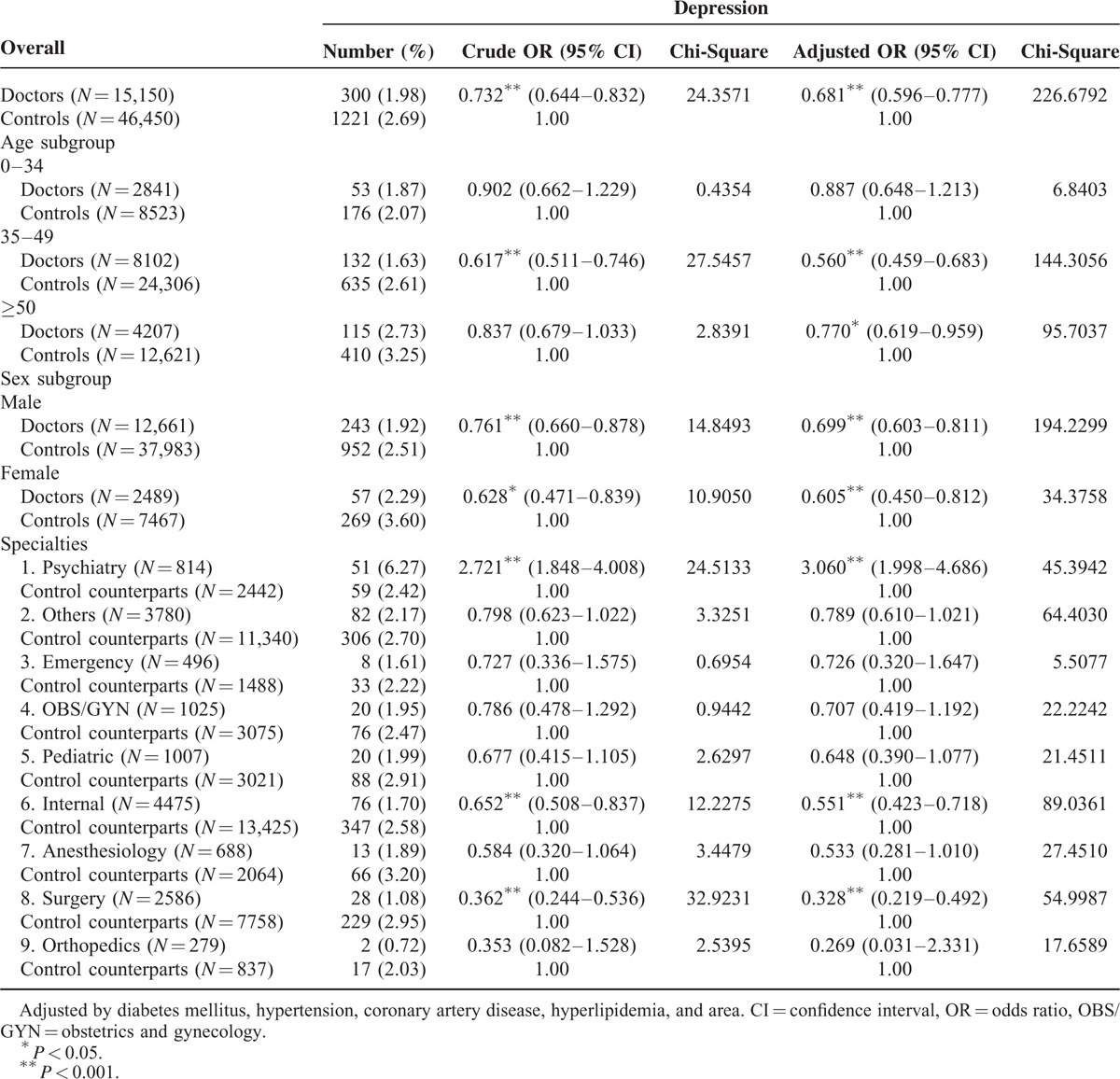
Stratified ORs for Depression by Age, Sex, and Specialty of Physicians

## DISCUSSION

This study aimed to investigate the actual risk for stress-related psychiatric problems in health care-seeking physicians using a large sample from a national representative database, the Taiwan's NHIRD. We designed a case–control study to evaluate the risk of treated insomnia, anxiety, and depression among hospital physicians by tracing their medical records for 2007 to 2011.

Overall, the results of this study suggest that physicians have lower odds of treated depression than the general population, although they have higher odds of treated insomnia and anxiety. These findings are partially in line with figures reported in the literature, which show that of 18% of Canadian physicians who were identified as depressed, only 25% considered getting help and only 2% actually did.^34^ Another study in Europe found that 78.3% of distressed hospital physicians had never sought professional help for depression/burnout.^30^ Gold et al^20^ used postmortem toxicology data and revealed that suicidal physicians were significantly more likely than nonphysicians to have antipsychotics, benzodiazepines, and barbiturates present on postmortem toxicology testing, but not antidepressants. Owing to the evidence of a higher prevalence of depression among physicians than among the general population,^8,9,11,14–16^ the undertreatment phenomenon may be due to reluctance or other barriers to help-seeking.

The risk of psychiatric problems in health care-seeking physicians seemed to vary among different specialties. Our data revealed that physicians in all specialties had significantly higher ORs for treated insomnia; among the highest was the emergency specialty. Psychiatry and “other” specialties had significantly higher odds of treated anxiety, but the OBS/GYN and surgery specialties had significantly lower odds of treated anxiety. As for depression, physicians in the psychiatry specialty had a significantly higher likelihood of depression treatment; internal and surgery specialties had significantly lower likelihood of depression treatment. Although these results partially echo those of some studies in which psychiatrists seemed to be highly stressed and burned out,^13,35,36^ there still is a possibility that physicians in other specialties were undertreated due to reluctance to deal with some barriers. A recent survey among members of the American College of Surgeons revealed that although 1 of 16 surgeons reported suicidal ideation in the previous year, only 26.0% with recent suicidal ideation had sought psychiatric or psychologic help.^4^ Another study in Europe also found that surgical specialists were least likely to have sought help.^30^ According to data from Wang et al,^15^ internal medicine doctors and surgeons had higher rates of depression than other specialties in Taiwan. Although having higher risks of psychiatric problems such as depression, undertreatment seemed more prevalent in some specialties of physicians.

It is worth noting that the risk of psychiatric problems in health care-seeking physicians may be influenced by sex and age. The present study found that male physicians had significant higher OR and female physicians had lower OR of treated anxiety. Moreover, there was no significant difference between likelihood of depression treatment between males and females. Empirical data indicate that the prevalence rates of anxiety, depression, or even suicide for female physicians were higher than those for male physicians^8,13,14,21^; however, another study showed a higher rate of anxiety or depression among male physicians.^11^ Our data did not support the findings that male physicians in Europe were less likely to have sought help.^30^

Another finding in the current study was that physicians age 35 to 50 years and >50 years had significantly lower ORs for treated depression than those age <35 years. This finding is in line with a previous study in which older, more experienced doctors reported less psychologic distress and burnout than younger doctors.^37^ Another study found that suicidal ideation among individuals ≥45 years was 1.5 to 3.0 times more common among surgeons than in the general population.^4^ Even though physicians in certain sex and age groups may be more vulnerable, they seemed not always to seek medical help.

The reasons why physicians are reluctant to seek help for behavioral health problems may be due mainly to the doctors’ attitudes. Research suggests that when faced with medical illness and psychologic distress, most physicians commonly use self-prescribed drugs and self-treatment.^16,27,28,30,38^ Doctors are reluctant to see another doctor, especially if the problem is psychologic.^4,31,32^ Furthermore, doctors get used to coping their stress or psychologic problem with denial and avoidance, although the effect is questionable.^25,26,29,39^ Moreover, a perceived stigma is related to resistance toward help-seeking. Doctors are not used to the role of patient, and fear that their need for help may present as an indicator of their weakness or inability to cope.^22,32,40^ It is worth noting that such fear of stigmatization may develop as early as medical school, when the physicians are students.^41^ Doctors’ attitudes can impede their access to appropriate health care for themselves. Results of the current study suggest that new approaches may be needed to reduce the stigma of psychiatric disorders, especially depression, and to enhance their prevention, detection, and treatment.

The present study has some limitations. The insomnia/anxiety/depression diagnoses used in the study were from administrative claims data reported by physicians according to ICD-9-CM, which may not be as valid as diagnoses made by structured interview. To increase the diagnostic validity, we enrolled only those individuals with insomnia/anxiety/depression who had at least 3 outpatient service claims within 1 year, or at least 1 inpatient hospitalization claim during study period. Another limitation is that our sample consisted hospital physicians only. Thus, caution needs to be taken when generalizing our findings to other physician group. Third, our study did not include substance use disorders, which is another important factor within physicians’ wellness.

In spite of these considerations, this study has valuable implications for practice and research. To the best of our knowledge, our study is the first to investigate the risk of common stress-related behavioral health problems in physicians, based on a nationwide population-based database. This study has added to the existing body of knowledge on sex, age, and specialty effects on health care-seeking for insomnia/anxiety/depression in physicians.

## CONCLUSIONS

Hospital physicians have lower odds of treated depression than the general population, although they have higher odds of treated insomnia and anxiety. Undertreatment was noted in some sex, age, and specialty groups of physicians. The reasons why physicians are reluctant to seek help for psychiatric problems need to be clarified in the future. Our study shed a light on the understanding the actual risk of common stress-related behavioral health problems in health care-seeking physicians. Doctor's mental wellness is a public health issue. Physicians suffering from these illnesses may deliver substandard care and may pose a possible threat to patients. These findings can be helpful to monitoring organizations and other Physician Health Programs related to physicians’ health promotion, monitoring, and licensing. Additional studies are needed to determine other factors affecting the doctors’ wellness such as substance use problem, how to reduce distress and burnout among doctors, how to improve detection of behavioral health problems in this population, and how to eliminate barriers to their use of psychiatry resources.
